# Inter-Network Brain Functional Connectivity in Adolescents Assigned Female at Birth Who Experience Gender Dysphoria

**DOI:** 10.3389/fendo.2022.903058

**Published:** 2022-07-22

**Authors:** Malvina N. Skorska, Nancy J. Lobaugh, Michael V. Lombardo, Nina van Bruggen, Sofia Chavez, Lindsey T. Thurston, Madison Aitken, Kenneth J. Zucker, M. Mallar Chakravarty, Meng-Chuan Lai, Doug P. VanderLaan

**Affiliations:** ^1^ Child and Youth Psychiatry, Centre for Addiction and Mental Health, Toronto, ON, Canada; ^2^ Brain Health Imaging Centre, Centre for Addiction and Mental Health, Toronto, ON, Canada; ^3^ Department of Medicine, Division of Neurology, Temerty Faculty of Medicine, University of Toronto, Toronto, ON, Canada; ^4^ Laboratory for Autism and Neurodevelopmental Disorders, Center for Neuroscience and Cognitive Systems @UniTn, Istituto Italiano di Tecnologia, Rovereto, Italy; ^5^ Department of Psychology, University of Toronto Mississauga, Mississauga, ON, Canada; ^6^ Department of Psychiatry, Temerty Faculty of Medicine, University of Toronto, Toronto, ON, Canada; ^7^ Cerebral Imaging Centre, Douglas Mental Health University Institute, Montreal, PQ, Canada; ^8^ Department of Psychiatry, McGill University, Montreal, PQ, Canada; ^9^ Department of Biological and Biomedical Engineering, McGill University, Montreal, PQ, Canada; ^10^ The Margaret and Wallace McCain Centre for Child, Youth & Family Mental Health and Azrieli Adult Neurodevelopmental Centre, Campbell Family Mental Health Research Institute, Centre for Addiction and Mental Health, Toronto, ON, Canada; ^11^ Department of Psychiatry and Autism Research Unit, The Hospital for Sick Children, Toronto, ON, Canada; ^12^ Department of Psychiatry, National Taiwan University Hospital and College of Medicine, Taipei, Taiwan; ^13^ Autism Research Centre, Department of Psychiatry, University of Cambridge, Cambridge, United Kingdom

**Keywords:** gender dysphoria, independent component analysis, resting-state fMRI, brain intrinsic functional organization, sexual orientation, adolescence, own-body perception

## Abstract

Gender dysphoria (GD) is characterized by distress due to an incongruence between experienced gender and sex assigned at birth. Brain functional connectivity in adolescents who experience GD may be associated with experienced gender (vs. assigned sex) and/or brain networks implicated in own-body perception. Furthermore, sexual orientation may be related to brain functional organization given commonalities in developmental mechanisms proposed to underpin GD and same-sex attractions. Here, we applied group independent component analysis to resting-state functional magnetic resonance imaging (rs-fMRI) BOLD timeseries data to estimate inter-network (i.e., between independent components) timeseries correlations, representing functional connectivity, in 17 GD adolescents assigned female at birth (AFAB) not receiving gender-affirming hormone therapy, 17 cisgender girls, and 15 cisgender boys (ages 12-17 years). Sexual orientation was represented by degree of androphilia-gynephilia and sexual attractions strength. Multivariate partial least squares analyses found that functional connectivity differed among cisgender boys, cisgender girls, and GD AFAB, with the largest difference between cisgender boys and GD AFAB. Regarding sexual orientation and age, the brain’s intrinsic functional organization of GD AFAB was both similar to and different from cisgender girls, and both differed from cisgender boys. The pattern of group differences and the networks involved aligned with the hypothesis that brain functional organization is different among GD AFAB (vs. cisgender) adolescents, and certain aspects of this organization relate to brain areas implicated in own-body perception and self-referential thinking. Overall, brain functional organization of GD AFAB was generally more similar to that of cisgender girls than cisgender boys.

## 1 Introduction

Gender dysphoria (GD) is defined as distress due to an incongruence between experienced gender and sex assigned at birth (1). A recent estimate of the population prevalence of GD is 0.5% in adolescents and adults (2). The number of adolescents referred clinically for GD, and particularly adolescents assigned female at birth (AFAB), has been steadily increasing over the past two decades (3, 4). Although this increase in GD adolescents has been reported, the neural basis of GD development continues to be understudied. The neural basis of GD is important to examine given that the most common scientific theories of the developmental origins of GD, which are discussed below, implicate brain development (e.g., 5–8). Furthermore, research regarding the neural basis of GD may provide insights into the neurodevelopmental processes associated with or underlying GD, which individuals who experience GD and their clinicians may find helpful in healthcare contexts (e.g., offering a neurobiological framework to understand why one experiences GD, 9).

The current study examined the intrinsic functional organization of the brain in GD AFAB adolescents not receiving puberty blockers or gender-affirming hormone therapy. Resting-state functional magnetic resonance imaging (rs-fMRI) and independent component analysis were used to estimate resting-state functional connectivity and to identify ‘networks’ with shared spatiotemporal patterns of activity. rs-fMRI is commonly used to examine functional connectivity of brain networks by investigating spatiotemporal correlations among the blood-oxygen-level dependent (BOLD) signals across the brain (10, 11). Research assessing functional connectivity using this methodology has found that brain regions demonstrate organized patterns of correlated BOLD signal changes, reflecting intrinsic operations of the brain (10–13). The brain regions with correlated BOLD activity may represent or even underpin networks associated with various broad domains of brain function (e.g., somatosensory, language, default mode, visual) (10–13).

One prior study examined functional connectivity in adolescents who experience GD. GD AFAB (*n* = 21; *M* age = 16.1 years, *SD* = 0.8) and GD assigned male at birth (AMAB; *n* = 19; *M* age = 15.4, *SD* = 1.1) adolescents, all receiving medication to suppress pubertal hormones, were compared with cisgender adolescents (*n* = 41) on functional connectivity within several networks selected *a priori* (14). In the right cerebellar hemispheric lobule VI within a visual network, GD AMAB showed stronger functional connectivity with the rest of that visual network compared to all other groups. In the right supplementary motor area within one of the sensorimotor networks, cisgender girls showed stronger functional connectivity with the other regions in that network compared with cisgender boys and GD AFAB; GD AMAB also showed stronger functional connectivity in this network than cisgender boys. In the right posterior cingulate gyrus, a component of the posterior default mode network, cisgender boys showed stronger functional connectivity to the rest of this network than all other groups. The authors interpreted the results as generally supporting two theoretical perspectives on brain characteristics in GD, which have also been supported by studies in GD adults (e.g., 15–18).

The first theoretical perspective posits that due to the incongruence between experienced gender and sex assigned at birth, GD individuals should show unique brain function patterns related to body perception and self-referential thinking (5, 15, 16, 19). Nota et al. (14) concluded that the visual network finding partly supported the idea that some aspects of brain function are unique to GD AMAB in the context of body perception. This conclusion was based on the pattern of group differences (i.e., GD AMAB > other groups) and on the cerebellar region and network underlying this group difference. Specifically, the cerebellum is involved in processing of negative emotional stimuli and GD individuals experience distress due to negative self-perception and psychosocial stress (20–22). Nota et al. (14) noted the experience of distress in GD individuals may influence functional connectivity in visual brain regions that are involved in emotion processing.

A second theoretical perspective is the neurohormonal hypothesis, which posits that prenatal androgen exposure organizes parts of the brain (in terms of structure (23) and function (24)) as well as sex-differentiated psychological and behavioral characteristics (25), including gender identity and sexual orientation. The surge in sex hormones during adolescence is argued to influence parts of the brain to be further expressed in a relatively male- or female-typical manner, based on the earlier prenatal brain organization (6–9, 26–28). On average, sex differences in functional connectivity that are consistent with the neurohormonal hypothesis have been reported (e.g., 13, 29–32), although there are ongoing debates regarding the extent of sex differences in various brain features, including functional connectivity (e.g., 33–36). In adolescents, sex differences were observed in the default mode, salience, sensorimotor, and executive control networks (14, 37, 38); however, the direction of effects is sometimes inconsistent and could be described by alternative theories (e.g., gender similarities hypothesis, 39, 40; the human brain mosaic, 41). The neurohormonal hypothesis predicts that, in GD, brain function should reflect the experienced gender due to variation in prenatal androgen exposure during prenatal brain development.

In Nota et al. (14), the idea that some brain function in GD adolescents reflects the experienced gender was partly supported by the sensorimotor network finding in both GD AFAB and GD AMAB, and the default mode network finding in GD AMAB. Again, this interpretation was made based on the pattern of group differences (e.g., cisgender boys > all other groups, including GD AMAB in the posterior default mode network). Also, this interpretation was made based on the regions and networks related to the group differences. For example, the posterior cingulate is involved in self-referential and spatial cognitive processes and sex differences in the default mode network have been shown (e.g., 37).

However, circulating sex hormones have also been related to patterns of functional connectivity (42–45). Puberty blockers and gender-affirming hormone therapy are often prescribed to GD adolescents to ameliorate GD and to promote gender transition, including the development of the desired physical appearance (8, 46, 47). Indeed, the GD adolescents in Nota et al. (14) were receiving puberty blockers. To test the neurohormonal hypothesis, it is therefore critical to examine brain function in GD *prior to* the initiation of puberty blockers or gender-affirming hormone therapy (6). The neurohormonal hypothesis has also been invoked to explain the development of sexual orientation (48–51), but sexual orientation has seldom been investigated in neurobiological studies of GD adolescents (6, cf. 17, 52 for studies in adults). For example, Nota et al. (14) did not examine associations with sexual orientation, likely because all GD AFAB adolescents were gynephilic (i.e., attracted to girls/women) and the GD AMAB adolescents were mostly androphilic (i.e., attracted to boys/men). In a recent study on cortical structure in GD AFAB adolescents, shorter T1 relaxation time (reflecting denser gray matter) was associated with older age and gynephilia in cisgender boys and GD AFAB, and with stronger attractions in cisgender boys (53). This study further highlighted the importance of investigating sexual orientation in brain studies of GD and the need to consider both the target(s) and strength of sexual attractions. In other words, sexual orientation is a separate construct from GD and is not core to the diagnosis or treatment planning of GD; however, it is an important confounding factor in the research on the neural basis of GD given some potentially overlapping developmental mechanisms proposed to underlie sexual orientation and GD.

The results of Skorska et al. (53), along with the wider literature on adolescent brain development, also emphasize the importance of examining age in studies of adolescent GD. The brain undergoes substantial changes during adolescence (e.g., 54, 55). The default mode, salience, visual, and sensorimotor networks develop earlier whereas executive control networks develop later (11, 55). Also, there is some evidence that within-network functional connectivity becomes weaker with age, whereas between-network functional connectivity strengthens with age, although there are some network-dependent discrepancies (11, 56, 57). Furthermore, both integration (increased long-range connections) and segregation (decreased short-range connections) in brain networks occur with age (58, 59). These findings underscore the need to consider age in functional connectivity research on adolescent GD, particularly regarding inter-network functional connectivity.

The present study examined inter-network (i.e., between independent components, ICs) functional connectivity in GD AFAB adolescents not receiving puberty blockers or gender-affirming hormone therapy relative to cisgender girls and boys. Given there is only one other rs-fMRI study published on GD adolescents to date (14), we used a data-driven approach, with the primary aim to identify multivariate associations in functional connectivity that distinguished the three groups. A secondary aim was to evaluate group differences in functional connectivity related to sexual orientation and age. Two aspects of sexual orientation were examined: degree of androphilia-gynephilia and strength of sexual attractions. The Nota et al. (14) study used an intra-network approach with the following limitations: only regions-of-interest from select networks were examined, network selection was based on the results of studies in GD adults, GD adolescents were receiving puberty blockers, and variation in sexual attractions was not examined. Thus, our rs-fMRI study is the first to examine in GD AFAB adolescents: (i) functional connectivity derived using a whole-brain, data-driven approach, which permitted the assessment of pair-wise inter-network connectivity across multiple common resting-state networks, (ii) an adolescent GD AFAB sample not receiving puberty blockers or gender-affirming hormone therapy, and (iii) sexual attractions alongside functional connectivity.

We were interested in how patterns of inter-network functional connectivity aligned with the two hypotheses of intrinsic brain organization in GD individuals. The hypothesis regarding own-body perception and self-referential thinking predicts that functional connectivity of GD AFAB should differ from both cisgender boys and girls. Also, group differences should be associated with regions aligning with own-body perception and self-referential thinking (e.g., visual networks and the cerebellum, as found in 14). The neurohormonal hypothesis predicts that functional connectivity in our GD AFAB group should be similar to that of cisgender boys and differ from cisgender girls. Further, functional connectivity patterns of gynephilic GD AFAB should reflect those of gynephilic cisgender boys.

## 2 Materials and Methods

### 2.1 Participants

Forty-nine adolescents participated in the study from 2014 to 2018: 17 AFAB adolescents with a DSM-5 diagnosis of GD who were not receiving puberty blockers or gender-affirming hormone therapy (mean (*M*) age = 184.73 months, *SD* = 24.69, range = 147-216), 17 cisgender girls (*M* age = 192 months, *SD* = 14.87, range = 162-216), and 15 cisgender boys (*M* age = 191.71, *SD* = 19, range = 152-214). GD AFAB were diagnosed via a clinician assessment. Participants were 12- to 17-years-old, with a mean age of 15.29 years (*SD* = 1.62). The majority were “European”/”White” (*n* = 29, 59.2%) and the remainder were other ethnicities (*n* = 20, 40.8%) (see **Supplementary Material**). All participants had begun puberty prior to the time of participation (see **Supplementary Material**). Data from two additional participants were removed: one GD AFAB adolescent due to artifacts from a metal orthodontic expander and one cisgender boy due to excessive head motion during T1-weighted (T1w) scans.

Forty-seven of the 49 adolescents in the current study were included in Skorska et al. (53). The difference in sample size was due to unusable T1 maps in two participants; these T1 maps were required to derive T1 relaxation time in Skorska et al. (53), but not required for functional connectivity analyses in the current study. Inclusion criteria, exclusion criteria, recruitment procedures, and general procedures were the same as in Skorska et al. (53) and those details can be found in the **Supplementary Material**. Only measures relevant to the aims of this study are reported. The study was conducted in accordance with the Declaration of Helsinki, and the protocol was approved by the Research Ethics Board of the Centre for Addiction and Mental Health (#145-2013).

### 2.2 Measures

All numerical values for variables reported and most of the code used are available from Borealis (60). For measures where a mean is calculated, the mean was computed for those participants who responded to at least 75% of the items.

#### 2.2.1 Age

Age in months was calculated by subtracting the date of birth from the date that the consent form was signed (rounded to the nearest month). The MRI scan was conducted on the same day as consent for 42 participants, within one month for six participants, and within two months for one participant.

#### 2.2.2 Gender Dysphoria

The Gender Identity/Gender Dysphoria Questionnaire for Adolescents and Adults (GIDYQ-AA) (61, 62) is a 27-item scale assessing gender identity and gender dysphoria with good discriminant validity and clinical utility (61–63). Cisgender boys completed the male version and cisgender girls and GD AFAB completed the parallel female version. Each item was rated with respect to the past 12 months on a 5-point Likert-type scale from 1 (always) to 5 (never). An example of a female version item is: “In the past 12 months, have you felt unhappy being a girl?” A mean score was calculated, with lower scores indicating greater gender dysphoria. Cronbach’s alpha was 0.98.

#### 2.2.3 Sexual Orientation

The Erotic Response and Orientation Scale (EROS) is a 16-item self-report measure of sexual orientation assessing sexual attractions and fantasies over the past 6 months with good discriminant validity (64–66). Eight questions pertain to attractions/fantasies toward boys/men (i.e., androphilia; e.g., “How often have you noticed you had any sexual feelings [even the slightest] while looking at a boy/man?”). The other eight questions used similar language to assess attractions/fantasies toward girls/women. Frequency of occurrence for each item was rated on a 5-point scale ranging from 1 (not at all) to 5 (almost every day). Mean androphilia and mean gynephilia scores were derived for each participant, where higher scores reflected more attractions/fantasies. Internal consistency on both scales was high (Cronbach’s alpha: androphilia = 0.94, gynephilia = 0.95).

EROS scores measure two aspects of sexual orientation: strength of attractions (i.e., none to many attractions, regardless of orientation) and degree of androphilia-gynephilia. To characterize these two aspects, two metrics were created for each participant, as in Skorska et al. (53). A summary is provided here and details, including the formulas used, can be found in Skorska et al. (53). A vector, $\vec{AndroGyne}$ , was created by calculating the strength (magnitude) and degree (phase, *θ*) of sexual attractions from the individual androphilia and gynephilia scores, which were not significantly correlated with each other (see Results). Based on EROS scores (ranging from 1 to 5 on each scale), the strength (magnitude) of sexual attractions is denoted by the length of the vector and degree of androphilia-gynephilia (phase) is denoted by the angle the vector creates with the x-axis. The magnitude and phase are symmetrically distributed ± 34° around 45° such that an 11° phase indicates exclusive androphilia, a 79° phase indicates exclusive gynephilia, and a 45° phase indicates equal scores on androphilia and gynephilia (e.g., asexual or ambiphilic).

### 2.3 MRI Methods

#### 2.3.1 Image Acquisition

All participants were scanned at CAMH in Toronto, Ontario, Canada on a GE MR750 3T magnetic resonance scanner (General Electric, Milwaukee, WI) with an 8-channel head coil (General Electric, 8HR BRAIN, GE Standard 8-Channel Head Coil). T1w images were acquired with a T1 BRAVO pulse sequence in the sagittal plane. The acquisition parameters were: inversion time = 650 ms, echo time = 3 ms, repetition time = 6.8 ms, flip angle = 8°, field of view = 23 cm, 256 x 256 matrix, 200 isotropic 0.9-mm thick slices, acquisition time = 4:42 min. Resting-state fMRI was acquired with a T2*-weighted SPIRAL in/out 2D gradient echo sequence in the axial plane (67). The acquisition parameters were: field of view = 22 cm, 210 functional volumes (64 x 64 x 31 slices), slice order = sequential, slice thickness = 5.0 mm (voxel size = 3.4375 mm x 3.4375 mm x 5.0 mm), TE = 30 ms, TR = 2000 ms, flip angle = 60°, acquisition time = 7 min. The relatively larger 5 mm slice thickness was chosen to ensure enough data points were acquired over 7 minutes given limited scan time. Participants were instructed to, “Keep your eyes closed, let your mind wander, and do not think about anything in particular. Do not fall asleep.”

#### 2.3.2 T1w Image Processing

To extract the brain from the raw T1w image, FSL’s (FMRIB Software Library, version 6.0.4, https://fsl.fmrib.ox.ac.uk/fsl/fslwiki/) BET (Brain Extraction Tool) was used with a fractional intensity threshold of 0.3.

#### 2.3.3 fMRI Processing

The spiral-in and spiral-out images were first denoised in the native space separately to remove potential spiral-related artifacts; no other artifacts were removed at this stage. During this initial denoising/“cleaning” process, realignment across volumes was done using FSL’s (version 5) MCFLIRT (Motion Correction FMRIB’s Linear Image Registration Tool) and brain extraction occurred using BET. Here, MELODIC (Multivariate Exploratory Linear Optimized Decomposition into Independent Components) was used to identify spiral-related artifacts, and FIX (FMRIB’s ICA-based X-noiseifier) and fsl_regfilt were used to sort and remove these artifacts, respectively. After removing spiral-related artifacts, the spiral-in and spiral-out images were combined using a weighted average (68). Next, FSL’s (version 6.0.4) fslreorient2std was used to re-orient the combined spiral image to match the orientation of the MNI-152 template. Then, the first volume of each re-oriented combined spiral image was removed using FSL’s fslroi because the contrast for this volume differed from the contrast in other volumes. Thus, a total of 209 volumes were included.

Next, single-session independent component analysis (ICA) using MELODIC (version 3.15) was run for each participant’s dataset in the native space. Default settings in the GUI were used, with the exception that any processing options already used in the above-mentioned spiral cleaning step were not reapplied: i.e., no brain extraction, no motion correction, and no additional volumes removed. Additionally, slice-timing correction was not applied and nonlinear registration was selected (to create the relevant files for nonlinear registration of the functional data to template; see below). The T1w images with and without the skull stripped were used to produce the files needed for nonlinear registration to template. For each individual dataset, noise components (i.e., pulsatility, remaining motion artifacts after realignment, other artifacts) were manually identified using Salimi-Khorshidi et al. (69) as a guide and removed using fsl_regfilt. The individually denoised data were then nonlinearly registered to MNI-152 space using FSL’s applywarp with output from the single-session ICA (i.e., the affine matrix for the transformation and the file with warp/coefficient information).

**Figure 1:**
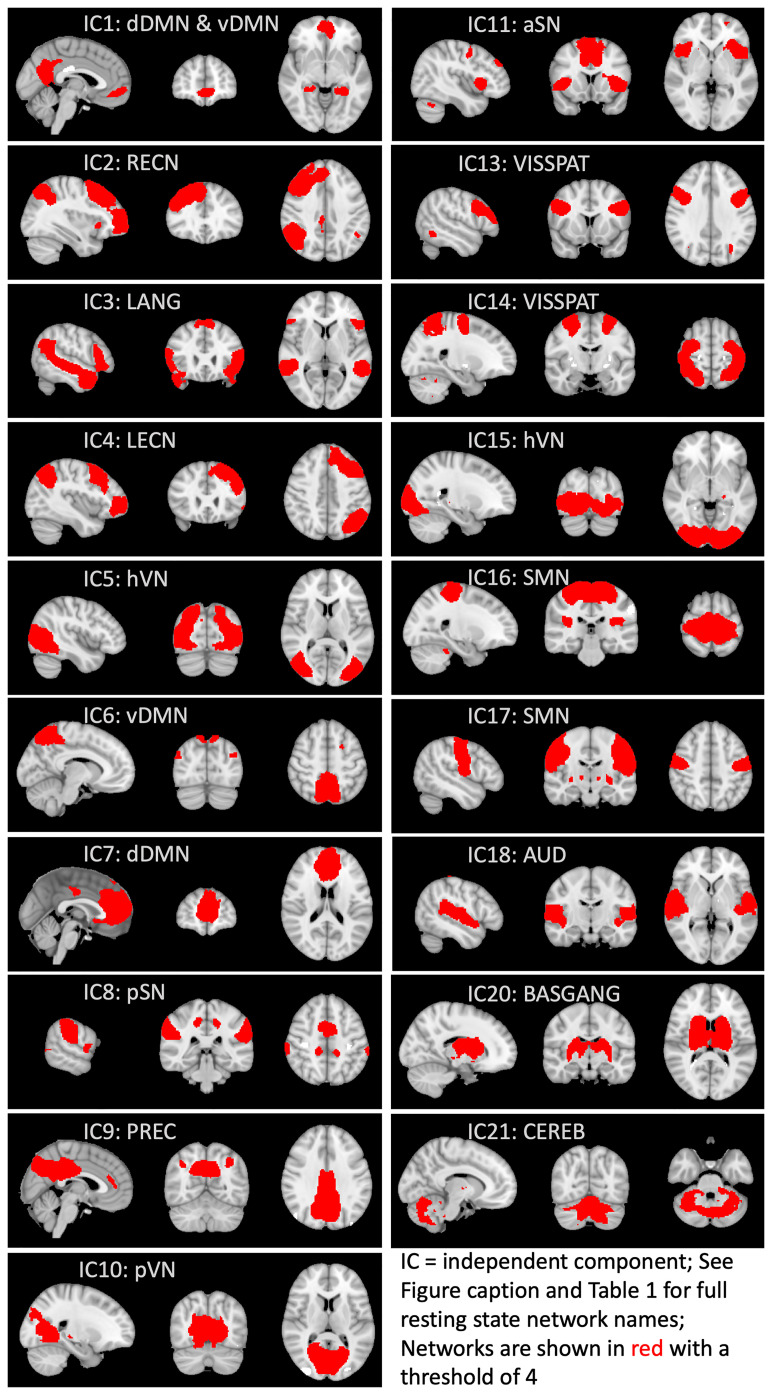
Functional connectivity networks derived from group independent component analysis. Network names are from the Find Lab atlas (71). Independent components (ICs) 12, 19, and 22-25 were discarded because they were primarily localized in white matter, did not reflect activity restricted primarily to gray matter, or represented noise/artifacts. DMN, default mode network; vDMN, ventral DMN; dDMN, dorsal DMN; RECN, right executive control network; LANG, language network; LECN, left executive control network; hVN, high visual network; pSN, posterior salience network; PREC, precuneus network; pVN, primary visual network; aSN, anterior salience network; VISSPAT, visuospatial network; SMN, sensorimotor network; AUD, auditory network; BASGANG, basal ganglia network; CEREB, cerebellum.

To delineate common resting-state networks (RSNs) across all participants, the denoised individual images in MNI-152 space were submitted to a group ICA using MELODIC, constraining the dimensionality to 25 ICs (independent components) (70) to focus on well-known large-scale networks (https://open.win.ox.ac.uk/pages/fslcourse/practicals/ica/index.html#gica). These 25 ICs accounted for 50.44% of the total variance (**Table 1**). Dual regression then back-projected the spatial maps and timeseries for each IC to generate participant-specific spatial maps and timeseries. From the group ICA, all ICs were visually examined to determine non-noise RSNs at the group level. Also, the spatial maps were checked against the Find Lab atlas (71) using FSL’s fslcc and a correlation threshold of 0.20 to examine the convergence of the RSNs in our sample with commonly identified RSNs in the literature (see **Supplementary Material**). Six ICs were discarded because they were primarily localized in white matter, did not reflect activity restricted primarily to gray matter, or represented remaining artifacts. Thus, 19 ICs were retained for the main analyses (**Table 1**; **Figure 1**).

**Table 1:** Independent components (ICs) from the Group Independent Component Analysis and Associated Resting-State Networks.

IC#	Network Name	% of Explained Variance	% of Total Variance
IC01	Dorsal default mode network (DMN) Ventral DMN	5.31	2.68
IC02	Right executive control network	5.07	2.56
IC03	Language network	4.92	2.48
IC04	Left executive control network	4.65	2.34
IC05	High visual network	4.61	2.33
IC06	Ventral DMN	4.55	2.30
IC07	Dorsal DMN	4.54	2.29
IC08	Posterior salience network	4.51	2.27
IC09	Precuneus network	4.51	2.27
IC10	Primary visual network	4.43	2.23
IC11	Anterior salience network	4.17	2.10
IC13	Visuospatial network	4.09	2.06
IC14	Visuospatial network	4.05	2.04
IC15	High visual network	4.02	2.03
IC16	Sensorimotor network	3.97	2.00
IC17	Sensorimotor network	3.65	1.84
IC18	Auditory network	3.64	1.84
IC20	Basal ganglia network	3.29	1.66
IC21	Cerebellum	3.10	1.56
	Total	81.08	40.88

Network names are from the Find Lab atlas (71). ICs 12 (EV, or % of explained variance = 4.12; TV, or % of total variance = 2.08), 19 (EV = 3.32, TV = 1.67), 22 (EV = 3.05, TV = 1.54), 23 (EV = 2.99, TV = 1.51), 24 (EV = 2.89, TV = 1.46), and 25 (EV = 2.57, TV = 1.30) were discarded because they were primarily localized in white matter, did not reflect activity restricted primarily to gray matter, or represented noise/artifacts. Explained variance including discarded ICs = 100% and total variance including discarded ICs = 50.44%.

To estimate inter-network functional connectivity between these ICs, timeseries for each participant and each of the 19 ICs were used to create inter-network correlation coefficients for each participant, similar to Bertelsen et al. (70). An in-house Matlab (2021a, version 9.10.0.1602886) (72) script using the *nets_netmats.m* function in the FSLNets Matlab toolbox was used to calculate a partial correlation matrix among the 19 ICs (see https://github.com/IIT-LAND/adir_subtyping/tree/master/code for original code; see Skorska et al. (60) for the code as amended for the current study). Ridge regression with a *rho* of 1 (i.e., using Tikhonov-regularization) was used to regularize the partial correlation coefficient for more robust estimates. Partial correlations removed indirect connections between the ICs to assess connection strengths more directly between network pairs. The partial correlation coefficients were then converted to *z*-statistics using Fisher’s *r*-to-*z* transformation and the lower diagonal of the *z*-scored partial correlation matrix was extracted. This produced 171 IC pairs for each participant. The *r*-to-*z* transformed partial correlations were used in partial least squares (PLS) analyses (see below). For figures showing IC-IC *z*-scores, these transformed correlations are shown averaged across all participants within each group.

### 2.4 Statistical Analyses

#### 2.4.1 Group Differences in Demographic and Psychosexual Variables

Using SPSS version 27 (IBM Corp., 2020), group differences for age, GIDYQ-AA, and EROS variables were examined with one-way analyses of variance (ANOVAs). In the presence of a significant omnibus effect, *post hoc* comparisons were conducted with least significant difference (LSD) tests. The GIDYQ-AA had a significant Levene’s test, so the Games-Howell *post hoc* test, robust to heterogeneity of variance, was used for this measure. A two-tailed critical level of 0.05 was used.

As reported in the **Supplementary Material**, we also investigated ethnicity, parent education, parent marital status, subtests on the Wechsler intelligence scales capturing verbal comprehension and visual spatial indices, pubertal development, regularity of menstrual cycle (in AFAB participants only), medication use, externalizing, internalizing, and overall mental health challenges. Also, correlations between these variables and the inter-network functional connectivity data were explored and Bonferroni-corrected for multiple comparisons. No demographic variable correlated with the functional connectivity data after correction, and age, ethnicity, parent education, verbal comprehension index, visual spatial index, pubertal development, regularity of menstrual cycle, and externalizing mental health challenges did not show significant group differences. Thus, apart from including age as a variable of interest in specific analyses, no other demographic variables were included in the main analyses.

#### 2.4.2 Partial Least Squares Analyses

The multivariate data-driven statistical technique partial least squares (PLS) (73, 74) was used to examine the relationship between the functional connectivity data and group. Specifically, PLS allows for examining the contribution of group to patterns of inter-network functional connectivity. PLS is well-suited for the analysis of inter-network functional connectivity data because it deals well with data that are highly correlated and in situations where the number of observations is substantially larger than the number of participants. PLS identifies distributed patterns in brain data, including functional connectivity data (75). The data matrix was created as a single row for each participant containing 171 partial correlations transformed to *z*-values. The results are referred to by an IC-pair designation (e.g., “IC1-IC4”).

The data matrix is factorized *via* singular value decomposition to produce latent variables with three components: a vector representing group and/or task contrasts (***v***), functional connectivity network pair saliences/weights (***u***), and a measure of the strength of that relationship (***s***). Permutation tests using 1000 permutations provided an exact probability and assessed the number of times the strength of the permuted latent variable exceeded the observed strength. Bootstrap resampling using 1000 samples estimated the standard error of each functional connectivity network pair salience to assess the reliability of each salience’s contribution to the observed pattern. The ratio of the salience to its standard error approximates a *z*-score, and a threshold of ± 2.5 was used to identify the most stable, reliable saliences. The bootstrap sampling distribution was also used to calculate 95% confidence intervals around the point estimates. “Brain scores” were the dot-product of the projection of the functional connectivity network pair saliences on each participant’s data, and indicated how strongly each participant reflected the contrast identified on a latent variable.

In the first PLS, we examined how the three groups (GD AFAB, cisgender boys, cisgender girls) contributed to an optimal combination of functional connectivity network pair associations. Age was not included in this analysis (i.e., we did not regress age out from the functional connectivity data) given no group differences in age and given no significant correlations between age and the functional connectivity data after correcting for multiple tests (see also Results and **Supplementary Material**). A second PLS identified the similarities and differences in correlations between the functional connectivity data and behavior variables across the three groups. The behavior variables were the two EROS variables (i.e., strength of attractions and degree of androphilia-gynephilia) and age. Age was included because the strength of attractions scores correlated with age (see Results). Both PLS analyses were conducted using the PLS software available at https://www.rotman-baycrest.on.ca/index.php?section=84 (v. 6.1311050) and MATLAB (2021a, version 9.10.0.1602886) (72).

## 3 Results

### 3.1 Demographic and Psychosexual Variables

Group differences in age, the GIDYQ-AA, and the two EROS variables were similar to Skorska et al. (53) given the two samples are largely the same. As a result, we summarize group differences below and present the details in the **Supplementary Material**. There were no extreme deviations from normality based on skewness and kurtosis values, which were less than |2|.

**Figure 2:**
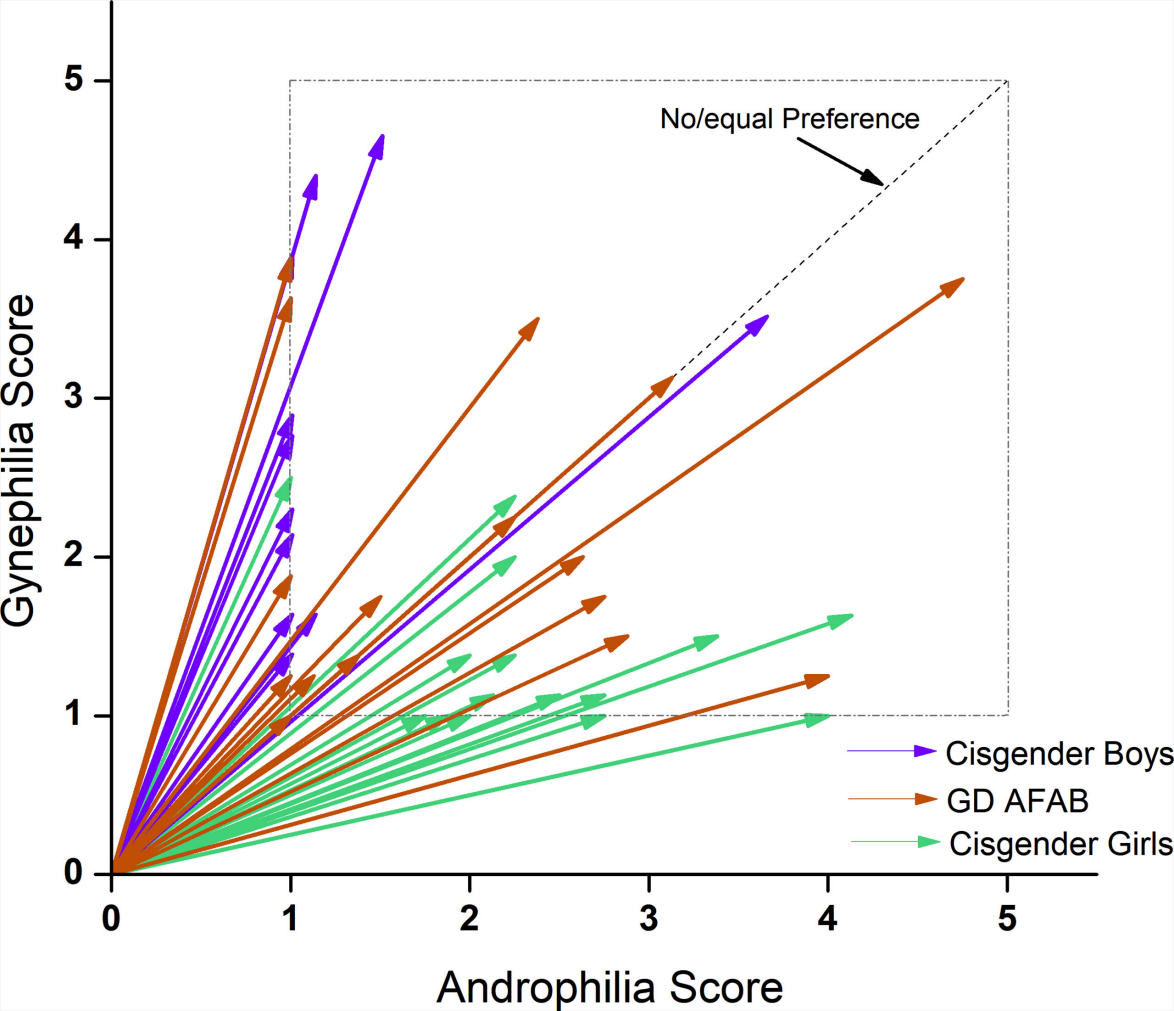
EROS scores by group. Cisgender girls (*n* = 17; green) were predominantly androphilic (< 45°), cisgender boys (*n* = 15; purple) were predominantly gynephilic (> 45°) and GD AFAB adolescents (*n* = 17; orange) expressed a range of sexual attraction targets.

Regarding age, the main effect of group was not significant. On the GIDYQ-AA, as expected, the GD AFAB group scored significantly lower than the cisgender girls and cisgender boys, who did not significantly differ from each other. For sexual orientation, there were no significant group differences on strength of attractions. For degree of androphilia-gynephilia, cisgender boys were more gynephilic than both GD AFAB and cisgender girls. GD AFAB were more gynephilic than cisgender girls. Cisgender boys tended to be gynephilic, cisgender girls tended to be androphilic, and GD AFAB had a range of sexual attractions with a cluster of individuals in the ambiphilic or asexual range (see **Figure 2**).

Androphilia scores were not related to gynephilia scores, and strength of attractions was not related to degree of androphilia-gynephilia across participants, nor within any group. Strength of attractions was significantly correlated with age across all participants and within cisgender boys and cisgender girls, but not within GD AFAB. Degree of androphilia-gynephilia was not significantly correlated with age across all participants, nor within any group.

### 3.2 PLS With Group and Functional Connectivity Data

**Figure 3:**
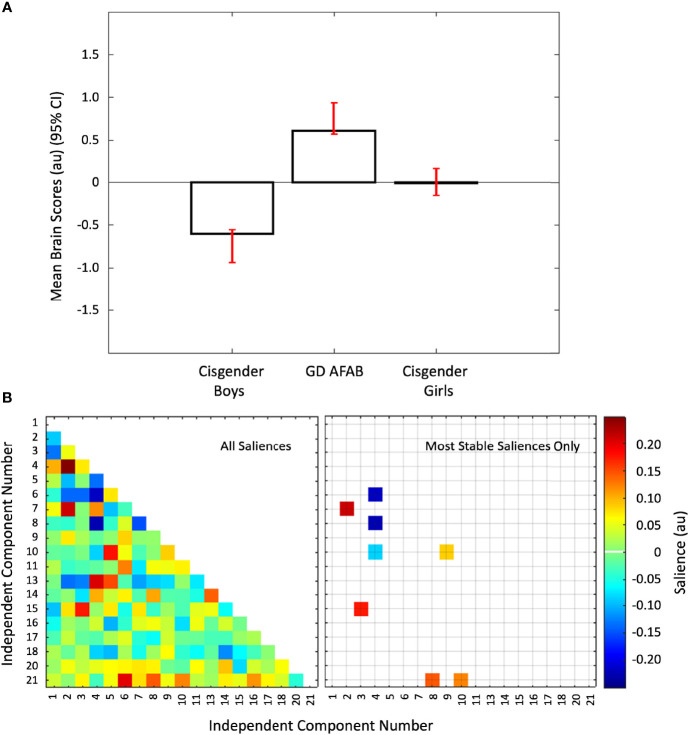
Group differences in inter-network functional connectivity. Panel **(A)** Mean brain scores (± 95% confidence interval, CI) from the first PLS analysis showing group differences: cisgender girls were intermediate between cisgender boys and GD AFAB (gender dysphoria, assigned female at birth). Scatterplots of brain scores by design scores for all participants are shown in **Figure S1** (**Supplementary Material**). Panel **(B)** Heat maps of the brain saliences for the 19 independent component (IC) pairs (left) and the eight IC pairs with the most stable saliences (right). The z-scored correlations within each group for the eight most stable IC pairs are displayed in **Figure S4** (**Supplementary Material**). ICs 12, 19, and 22-25 were discarded as noise or non-gray matter signal. Network names are in **Table 1**.

The first PLS analysis identified one significant latent variable (*P* = 0.043) that accounted for 58.77% of the covariance between group and the functional connectivity data. The other latent variable was not significant. This significant latent variable differentiated all three groups from each other such that cisgender girls were intermediate between GD AFAB and cisgender boys. Thus, the overall pattern partly reflected a sex-assigned-at-birth difference, with GD AFAB more strongly differentiated from cisgender boys than cisgender girls (**Figure 3**, Panel **A**). Five positive and three negative brain saliences were considered to be stable (**Figure 3**, Panel **B**). The networks associated with group differences were: IC10 (primary visual network) with IC09 (precuneus network) and IC21 (cerebellum); IC02 (right executive control network) with IC07 (dorsal default mode network); IC03 (language network) with IC15 (higher visual network); IC08 (posterior salience network) with IC21 (cerebellum); IC04 (left executive control network) with IC06 (ventral default mode network), IC08 (posterior salience network), and IC10 (primary visual network) (**Figure 3**, Panel **B**). Thus, the groups were distinguished by differences in correlation strength in functional connections between the left executive control network and the ventral default mode and posterior salience networks, as well as differences in functional connections involving the visual networks with the language, left executive control, precuneus, and cerebellum networks (**Supplementary Figure S4, Panel A**).

### 3.3 PLS With Group, Age, Sexual Orientation, and Functional Connectivity Data

**Figure 4:**
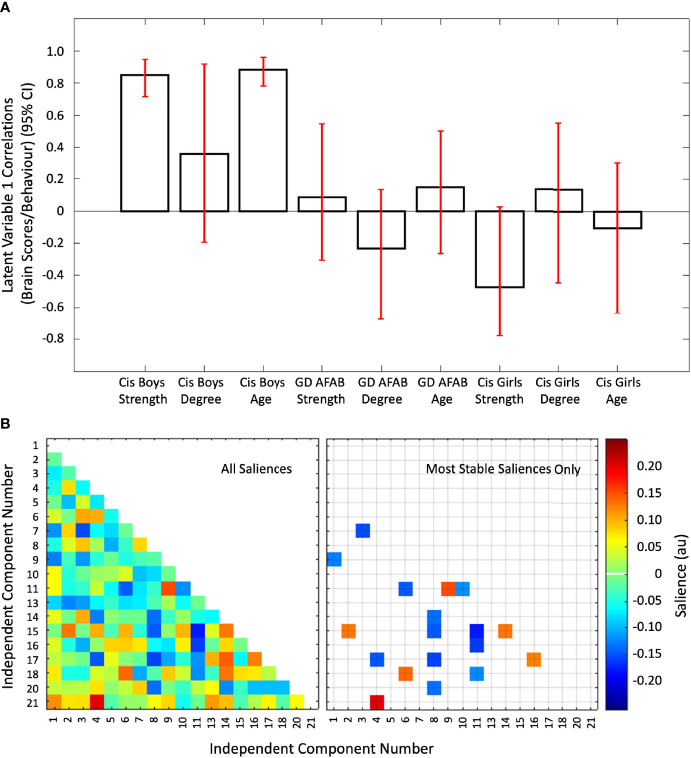
The first latent variable reflecting correlations of inter-network functional connectivity with age, strength of sexual attractions, and degree of sexual attractions. Panel **(A)** Cis = cisgender, Strength = strength of sexual attractions, Degree = degree of androphilia-gynephilia. Correlations of brain scores with behavior (± 95% confidence interval, CI) from the first significant latent variable in the second PLS analysis. This latent variable identified stable correlations in cisgender boys related to age and strength of sexual attractions. No correlations were stable in the cisgender girls or GD AFAB (gender dysphoria, assigned female at birth) adolescents. Scatterplots of brain scores by behavioral measures for all participants can be found in **Figure S2** (**Supplementary Material**). Panel **(B)** Heat maps of the brain saliences (left) and stable saliences (right) for 19 independent component (IC) pairs. This latent variable included stable positive and negative saliences in 18 IC pairs, shown on the right. ICs 12, 19, and 22-25 were discarded as noise or non-gray matter signal. Network names are in **Table 1**.

The second PLS analysis identified two significant latent variables. The other latent variables were not significant. The first latent variable (*P* = 0.006) accounted for 28.38% of the cross-block covariance between the functional connectivity data and the two EROS variables and age across the groups. The first latent variable was driven by stable correlations of age and strength of attractions with inter-network functional connectivity only in the cisgender boys (**Figure 4,** Panel **A**). There were six positive 12 negative brain saliences that were considered stable (**Figure 4**, Panel **B**). The networks involved were: IC15 (higher visual network) with IC02 (right executive control network) and IC14 (visuospatial network); IC04 (left executive control network) with IC17 (sensorimotor network) and IC21 (cerebellum); IC06 (ventral default mode network) with IC18 (auditory network); IC09 (precuneus network) with IC01 (dorsal and ventral default mode network) and IC11 (anterior salience network); IC16 (sensorimotor network) with IC17 (sensorimotor network); IC08 (posterior salience network) with IC14 (visuospatial network), IC15 (high visual network), IC17 (sensorimotor network), and IC20 (basal ganglia network); IC11 (anterior salience network) with IC06 (ventral default mode network), IC10 (primary visual network), IC15 (high visual network), IC16 (sensorimotor network), and IC18 (auditory network); and IC03 (language network) with IC07 (dorsal default mode network). In summary, strength of sexual attractions and age in cisgender boys were associated with a mix of strong and weak connections between the anterior salience network with high visual, primary visual, sensorimotor, ventral default mode, auditory, and precuneus networks, coupled with connections between the posterior salience network with visuospatial, basal ganglia, high visual, and sensorimotor networks (**Supplementary Figure S4**, Panel **B**).

**Figure 5:**
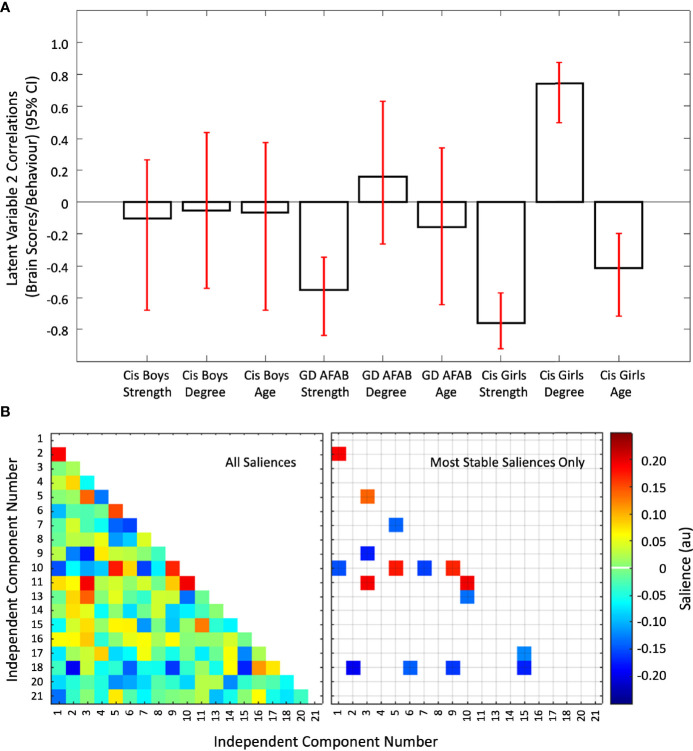
The second latent variable reflecting correlations of independent component pairs with age, strength of sexual attractions, and degree of sexual attractions. Panel **(A)** Cis = cisgender, Strength = strength of sexual attractions, Degree = degree of androphilia-gynephilia. Correlations of brain scores with behavior (± 95% confidence interval, CI) from the second significant latent variable in the second PLS analysis. This latent variable identified stable correlations in cisgender girls and GD AFAB (gender dysphoria, assigned female at birth). For cisgender girls, correlations were stable for strength of sexual attractions, age, and degree of androphilia-gynephilia. No correlations were stable in the cisgender boys and only strength of attractions was stable in GD AFAB participants. Scatterplots of brain scores by behavioral measures for all participants can be found in **Figure S2** (**Supplementary Material**). Panel **(B)** Heat maps of the brain saliences (left) and stable saliences (right) for 19 independent component (IC) pairs. This latent variable included stable positive and negative saliences in 16 IC pairs, shown on the right. ICs 12, 19, and 22-25 were discarded as noise or non-gray matter signal. Network names are in **Table 1**.

The second latent variable (*P* = 0.010) accounted for the next 18.32% of the cross-block covariance in this dataset. This latent variable was driven by stable correlations of age, strength of attractions, and degree of androphilia-gynephilia in cisgender girls, with GD AFAB showing a similar pattern for strength of attractions only (**Figure 5**, Panel **A**). There was a mixture of six positive and 10 negative saliences that were considered most stable (**Figure 5**, Panel **B**). The networks involved were: IC03 (language network) with IC05 (high visual network), IC09 (precuneus network), and IC11 (anterior salience network); IC10 (primary visual network) with IC01 (dorsal and ventral default mode network), IC05 (high visual network), IC07 (dorsal default mode network), IC09 (precuneus network), IC11 (anterior salience network), and IC13 (visuospatial network); IC01 (dorsal and ventral default mode network) with IC02 (right executive control network); IC18 (auditory network) with IC02 (right executive control network), IC06 (ventral default mode network), IC09 (precuneus network), and IC15 (high visual network); IC05 (high visual network) with IC07 (dorsal default mode network); and IC15 (high visual network) with IC17 (sensorimotor network). Overall, in cisgender girls, strength of sexual attractions, degree of androphilia-gynephilia, and age, and strength of attractions in GD AFAB were associated with weaker connections between the primary visual network with default mode, visuospatial, precuneus, high visual, and anterior salience networks, coupled with connections between the high visual network with auditory, dorsal default mode, sensorimotor, and language networks (**Supplementary Figure S4**, Panel **C**).

## 4 Discussion

Using a data-driven, multivariate approach, we examined group differences between adolescent GD AFAB, cisgender boys, and cisgender girls in inter-network brain functional connectivity, as well as associations between inter-network connectivity and sexual attractions and age. We found that inter-network functional connectivity in GD AFAB adolescents naïve to puberty blockers and gender-affirming hormone therapy differed from both cisgender girls and cisgender boys, who also differed from each other. This pattern suggests a sex-assigned-at-birth effect in cisgender adolescents, with a functional connectivity pattern that also differs from GD AFAB. Thus, the prediction that the brain intrinsic functional connectivity of GD AFAB is similar to their experienced gender (cisgender boys) was not supported; however, the prediction that the brain intrinsic functional organization of GD AFAB differs from both cisgender boys and girls was supported. Two patterns reflected sex-assigned-at-birth differences in the associations between brain functional connectivity, sexual orientation, and age: the first driven by cisgender boys and the second driven by individuals with a female sex-assigned-at-birth. Further, the relationship between functional connectivity and sexual orientation indicated that GD AFAB shared some features with cisgender girls. This pattern did not support the prediction that the brain functional connectivity patterns of gynephilic GD AFAB should reflect those of gynephilic cisgender boys. Next, we detail and interpret these patterns in the context of the wider literature.

Studies of cisgender adolescents and adults show a sex-assigned-at-birth difference in functional connectivity across several resting-state networks (e.g., 13, 29–32). Specifically, in adolescents, sex differences in the default mode, salience, sensorimotor, and executive control networks have been found (14, 37, 38). Therefore, our finding of a sex-assigned-at-birth difference, especially between the cisgender participants, aligns with previous studies. Furthermore, some of the networks underlying the sex difference in the current study have also been found in the broader literature. For example, we found that connectivity between the right executive control network and the dorsal default mode network was part of the pattern reflecting a sex difference. Also, the sex difference pattern involved connectivity between the left executive control network with the ventral default mode network and the posterior salience network. Similar networks showed sex differences in Muller-Oehring et al. (37) and Teeuw et al. (38). Our findings go beyond these previously reported findings of global network sex-assigned-at-birth differences by showing that inter-network interactions involving multiple network pairs also evidence sex-assigned-at-birth differences in functional connectivity.

Regarding GD AFAB adolescents, our finding that their brain functional connectivity differed compared with cisgender boys and girls partly aligns with the only other study on functional connectivity in GD adolescents. Nota et al. (14) found that GD AMAB showed stronger functional connectivity than GD AFAB, cisgender girls, and cisgender boys (who did not differ from each other) in the right cerebellar hemispheric lobule VI within the visual network. Although the pattern in Nota et al. (14) did not include GD AFAB, across both studies there is some support for the idea that functional connectivity in GD adolescents differs from that of cisgender adolescents. Furthermore, we found that part of the pattern of functional connectivity involved visual networks (i.e., primary visual network with the precuneus network and with the left executive control network, the language network with the higher visual network), the cerebellum (i.e., posterior salience network with the cerebellum), and involved functional connectivity between the primary visual network and the cerebellum. These patterns, particularly the connectivity between the primary visual network and the cerebellum, align with the finding reported in Nota et al. (14) in GD AMAB. Yet, unlike the present study, Nota et al. (14) found support for the idea that the functional connectivity of GD AFAB was similar to the experienced gender. Notable differences between our study and Nota et al. (14) include that we did not have a sample of GD AMAB, the participants in our study were not on puberty blockers (or gender-affirming hormone therapy), and we operationalized functional connectivity as between-IC rather than within-IC correlations. These methodological differences may explain the discrepancies in findings across studies.

Despite some of the differences across the studies, the networks involved in the group differences in Nota et al. (14) and in the current study (i.e., pairwise connections between visual, cerebellum, executive control, language, default mode, and salience networks) are in line with the hypothesis that functional connectivity differences in GD individuals are related to own-body perception and self-referential thinking. GD individuals experience an incongruence between the experienced gender and sex assigned at birth, which can include body incongruence. Thus, GD individuals may experience their bodies as not part of “self” and this may be reflected in brain functional connectivity (5, 16, 76). Many brain areas are involved in the complex integration of the bodily “self,” such as the precuneus, insula, posterior cingulate, inferior parietal lobule, primary somatosensory cortex, medial prefrontal cortex, fusiform gyrus, medial premotor cortex, angular gyrus, and pregenual anterior cingulate cortex, although the exact mechanisms remain unclear (5, 15, 16, 77, 78). These areas are also implicated in our findings; for example, the precuneus and the posterior cingulate as part of the default mode network, the insula as part of the salience network, and the angular gyrus as part of the executive control and language networks (71). Thus, our results are in line with the own-body perception and self-referential thinking hypothesis of GD.

When brain functional connectivity was assessed in relation to age and sexual orientation, the findings mostly reflected effects related to age and strength of sexual attractions. The suites of brain networks involved in these associations differed based on sex assigned at birth. Specifically, connectivity between the default mode network and the anterior salience network, the precuneus network, and the language network contributed to the pattern in cisgender boys, whereas connectivity between the default mode network and the primary visual network, high visual network, and the auditory network contributed to the pattern in cisgender girls and GD AFAB. Thus, these patterns can be interpreted as a sex-assigned-at-birth difference for functional connectivity related to age and sexual attraction strength during adolescence. The observed age effect is in line with previous research showing that, for at least some network connections, inter-IC functional connectivity strengthens with age (11, 56, 57).

In contrast, brain correlates of the strength of sexual attractions—and mechanisms underlying the development of this aspect of sexual orientation—are understudied. With age, the intensity of sexual behaviors increases (e.g., from hugging to having sexual intercourse) in cisgender boys and girls (79). To our knowledge, insights regarding how such increases in sexual motivation relate to adolescent brain development are limited to those reviewed here and in our recent study of largely overlapping participants with the present study (53). In that study, shorter T1 relaxation time, reflecting denser gray matter, was associated with older age and gynephilia in cisgender boys and GD AFAB adolescents, and with stronger attractions in cisgender boys (53). In contrast, here we found that functional connectivity was related to strength of attractions in cisgender boys as well as in cisgender girls and GD AFAB, but in different sets of brain networks depending on sex assigned at birth. Thus, our findings across the two studies point to sex- and gender-based differences in how sexual and brain development relate to one another and, furthermore, warrant future research examining associations between adolescent sexual development and various brain metrics among sexually and gender diverse individuals.

Although none of the findings indicated associations between functional connectivity and target of sexual attractions in GD AFAB participants, future research examining brain features of individuals who experience GD should nevertheless continue to consider this variable. As noted, in GD AFAB adolescents, shorter T1 relaxation reflecting denser gray matter appears to be associated with gynephilia (53). Moreover, with respect to functional connectivity, there is some evidence to suggest that the target of sexual attractions is relevant. Here, we found that degree of androphilia-gynephilia was associated with functional connectivity among cisgender girls (i.e., the second latent variable in the second PLS analysis; **Figure 5**). Of note, this pattern involved the precuneus and default mode network, which aligns with a prior study of adults that found that in the precuneus, which is part of the default mode network, gay men (androphilic AMAB) had less pronounced functional connectivity than lesbian women (gynephilic AFAB) (80). Such findings suggest target of sexual attractions could be relevant to certain brain features and, thus, should be investigated further in brain studies of GD despite the null findings regarding this variable reported here for GD AFAB.

### 4.1 Limitations

Caution is warranted in interpreting the present results given the caveats of neuroimaging research with small sample sizes (81). An additional limitation is that causal relationships cannot be determined given the cross-sectional, non-experimental design of the current study. We cannot infer whether differences in functional connectivity influence GD, gender identity, and/or sexual orientation, or vice versa. Furthermore, a convenience sample was used, which affects representativeness. We cannot fully disentangle experiences of distress, stigma, and mental health challenges that are associated with GD to examine functional connectivity only associated with an incongruence between sex-assigned-at-birth and experienced gender. This is because experiences of distress, stigma, and mental health challenges are commonly experienced by GD youth; to attempt to clarify this complexity, future research is needed using appropriate comparison groups (e.g., cisgender adolescents experiencing similar levels of distress, stigma, and mental health challenges). Finally, we did not directly examine behavioral indices related to own-body perception to interpret the results (e.g., 5).

GD AMAB adolescents were not included in the present study, and thus it is an open question whether the results can inform functional connectivity in GD AMAB adolescents. Also, generalization of our results to the broader sexual orientation population is not fully possible because there was only a small number of cisgender same-sex attracted adolescents in this sample. Our findings in the second PLS analysis may have been impacted by the restricted range in degree of androphilia-gynephilia in cisgender boys and cisgender girls. Furthermore, our results may not generalize to ages beyond 12-17 years and we are not able to robustly model developmental changes in functional connectivity given the cross-sectional design and limited sample size. Examining functional connectivity changes *via* longitudinal study designs with larger samples would provide more definitive insights regarding development. Last, we operationalized functional connectivity as inter-IC correlations, but functional connectivity has also been examined *via* other methods (e.g., intra-IC correlations, between-region connectivity based on *a priori* parcellation, functional connectivity represented using mutual information). These other processing methods and using fMRI data with higher spatiotemporal resolution may provide additional insights into brain functional organization related to GD.

### 4.2 Conclusion

In a sample of GD AFAB adolescents not receiving puberty blockers or gender-affirming hormone therapy, cisgender girls, and cisgender boys, a sex-assigned-at-birth difference was found in inter-network functional connectivity between the cisgender adolescents, with GD AFAB adolescents further differing from the cisgender adolescents. This pattern and the networks involved were consistent with the interpretation that the brain functional organization of GD AFAB differs from that of their cisgender peers. Furthermore, some of the patterns and networks observed were in line with the notion that GD reflects some alteration in functional connectivity in brain networks that could be involved in own-body perception and self-referential thinking. In the context of sexual orientation and age, the pattern and related brain networks in GD AFAB adolescents were similar to cisgender girls and differed from cisgender boys. Thus, the findings suggested adolescence is an important period of development of brain functional organization that correlates with age and sexual attractions for each sex—albeit with sex-assigned-at-birth differences in the functional networks involved.

## Data Availability Statement

The datasets and code for this study can be found in Borealis, the Canadian Dataverse Repository at https://borealisdata.ca/dataset.xhtml?persistentId=doi:10.5683/SP3/VQG2X6.

## Ethics Statement

The study was reviewed and approved by the Research Ethics Board (REB) of the Centre for Addiction and Mental Health (#145-2013).

## Author Contributions

Conceptualization, MS, NL, ML, NB, SC, KZ, MC, M-CL, and DV. Methodology, MS, NL, ML, KZ, MC, M-CL, and DV. Software and Resources, NL, ML, SC, and M-CL. Formal Analysis, MS, SC, NL, and M-CL. Data Curation, MS, NB, LT, and DV. Writing – Original Draft Preparation, MS, NL, M-CL, and DV. Writing – Review & Editing, MS, NL, ML, NB, SC, LT, MA, KZ, MC, M-CL, and DV. Visualization, MS, and NL. Supervision, NL, SC, MA, M-CL, and DV. Funding Acquisition, MS, NL, SC, KZ, MC, M-CL, and DV. All authors contributed to the article and approved the submitted version.

## Funding

MS was funded by Postdoctoral Fellowships from Brain Canada-Kids Brain Health Network, the Canadian Institutes of Health Research (CIHR), and the Discovery Fund from the Centre for Addiction and Mental Health (CAMH). This research was funded by CIHR Project Grants: [FRN 142348] awarded to NL, SC, KZ, MC, and DV, and [FRN 159578] awarded to MS, NL, MC, M-CL, and DV. M-CL was additionally funded by a CIHR Sex and Gender Science Chair [GSB 171373], the Academic Scholars Award from the Department of Psychiatry, University of Toronto, and the CAMH Foundation.

## Conflict of Interest

The authors declare that the research was conducted in the absence of any commercial or financial relationships that could be construed as a potential conflict of interest.

## Publisher’s Note

All claims expressed in this article are solely those of the authors and do not necessarily represent those of their affiliated organizations, or those of the publisher, the editors and the reviewers. Any product that may be evaluated in this article, or claim that may be made by its manufacturer, is not guaranteed or endorsed by the publisher.
