# Supplementary material for: Inter-Network Brain Functional Connectivity in Adolescents Assigned Female at Birth Who Experience Gender Dysphoria
Source: Front Endocrinol (Lausanne). 2022 Jul 22;13:903058. doi: 10.3389/fendo.2022.903058 (PMC9353716; doi:10.3389/fendo.2022.903058)
Supplement: Supplementary file 1 [file DataSheet_1.pdf]

Supplementary Material

Title: Inter-network brain functional connectivity in adolescents assigned female at birth who experience gender dysphoria

Authors: Malvina N. Skorska, Nancy J. Lobaugh, Michael V. Lombardo, Nina van Bruggen, Sofia Chavez, Lindsey T. Thurston, Madison Aitken, Kenneth J. Zucker, M. Mallar Chakravarty, Meng-Chuan Lai, & Doug P. VanderLaan

1 Recruitment Procedures

Recruitment procedures were the same as in Skorska et al. (1). Inclusion criteria were: age 12 to 17 years; and GD AFAB participants had to have received a diagnosis of GD via clinician assessment. For all participants, exclusion criteria were: receiving any form of hormone therapy (apart from oral contraceptive pills), a known disorder of hormone regulation or sex development, any contraindications to MRI (e.g., braces, pregnancy), experience of any head trauma, or insufficient English language proficiency to complete study measures. For cisgender participants, additional exclusion criteria were: a previous mental health diagnosis, being in a special education class in school, involvement with a child protection agency, or feeling uncomfortable with their sex assigned at birth and identifying or wishing to identify as a member of another gender.

GD AFAB participants were recruited from the Gender Identity Service (GIS) at the Centre for Addiction and Mental Health (CAMH) or were referred by a clinician in private practice specializing in GD. At the GIS, all prospective participants that were 12-17 years old were informed of the study. If they were interested, a member of the study team (not involved in their clinical care) shared additional information about the study, answered questions, performed screening, and obtained informed assent and/or consent to participate. Advertisements that described the study as being about brain development and gender were used to recruit cisgender participants from the community. These advertisements were posted online (Kijiji, *www.kijiji.ca*; Facebook *www.facebook.com*) and on bulletin boards in the Greater Toronto Area. Also, to facilitate snowball sampling, study information was distributed to cisgender participants via word of mouth.

Eligibility screening occurred in person or over the phone. For eligible participants, those between 12- and 15-years-old provided verbal assent and a parent/guardian provided informed consent, whereas those 16- to 17-years-old provided informed consent. The study consisted of: providing a 20-ml blood sample to measure hormones and other indices of physiological functioning, a 1-hour MRI session, a brief intelligence assessment, and completing a questionnaire package. Data were obtained from the clinical record for any GD participants who completed any study measures during a clinical assessment. All study procedures were completed on the same day for most participants, and the order of study parts was allowed to vary. Participants were thanked, received an honorarium of $20 CAD per hour or part thereof, and were compensated for travel expenses.

2 Group Differences in Demographic and Psychosexual Variables

Group differences in age, the GIDYQ-AA, and the two EROS variables were similar to Skorska et al. (1). A summary of results is in the main text and details are found in this section. Regarding age, the main effect of group was not significant (Table S1). There was a significant effect for the GIDYQ-AA such that the GD AFAB group scored significantly lower than the cisgender girls (mean difference [MD] = -2.71, *SE* = 0.09, $p < 0.001$) and cisgender boys (MD = -2.72, *SE* = 0.09, $p < 0.001$), who did not differ from each other (MD = 0.02, *SE* = 0.05, $p = 0.927$). The threshold for a potential diagnosis of GD is GIDYQ-AA < 3.00 (Zucker et al., 2012). The lowest score on the GIDYQ-AA from the cisgender participants was 4.48, indicating that none of the cisgender participants met this threshold; the highest score in the GD AFAB group was 3.04, with all other GD AFAB participants scoring below the threshold.

A main effect of group was found for degree of androphilia-gynephilia, whereas strength of attractions showed no significant group differences (Table S1). For degree of androphilia-gynephilia, cisgender boys were more gynephilic than both GD AFAB (MD = 17.78, *SE* = 4.51, $p < 0.001$) and cisgender girls (MD = 33.78, *SE* = 4.51, $p < 0.001$). GD AFAB were more gynephilic than cisgender girls (MD = 16.00, *SE* = 4.37, $p < 0.001$).

Androphilia scores were not significantly correlated with gynephilia scores across participants ($r = -0.08$, $p = 0.609$, $n = 49$), within cisgender boys ($r = 0.44$, $p = 0.105$, $n = 15$), within GD AFAB ($r = 0.26$, $p = 0.324$, $n = 17$), or within cisgender girls ($r = -0.16$, $p = 0.553$, $n = 17$). Strength of attractions was also not significantly correlated with degree of androphilia-gynephilia across participants ($r = 0.04$, $p = 0.762$, $n = 49$), within cisgender boys ($r = 0.22$, $p = 0.422$, $n = 15$), within GD AFAB participants ($r = -0.11$, $p = 0.667$, $n = 17$), or within cisgender girls ($r = -0.35$, $p = 0.163$, $n = 17$). Strength of attractions was significantly correlated with age across all participants ($r = 0.50$, $p < 0.001$, $n = 49$), within cisgender boys ($r = 0.87$, $p < 0.001$, $n = 15$) and cisgender girls ($r = 0.58$, $p = 0.014$, $n = 17$), but not within GD AFAB ($r = 0.09$, $p = 0.726$, $n = 17$). Degree of androphilia-gynephilia was not significantly correlated with age across all participants ($r = -0.01$, $p = 0.953$, $n = 49$), nor within any group: cisgender boys ($r = 0.39$, $p = 0.153$, $n = 15$), GD AFAB ($r = 0.16$, $p = 0.536$, $n = 17$), or cisgender girls ($r = -0.01$, $p = 0.958$, $n = 17$).

3 Examination of Additional Demographic Variables as Potential Covariates

Additional analyses were conducted to characterize the sample on demographic variables not presented in the main text and to examine whether these variables should be used as statistical controls in the main analyses.

3.1 Measures

The same measures were used as in Skorska et al. (1). A summary of measures and full results are presented below given some minor discrepancies in the results. Please refer to Skorska et al. (1) for methodological details not mentioned here.

Participants self-reported their ethnicity, education of their mother and father, and parents' marital status and number of years and months that the father has not been living at home (if the father was not living at home). Responses were dichotomized as in Skorska et al. (1) (see also Table S2). Subtests from the Wechsler Intelligence Scale for Children (version V) (2) were administered to participants 12- to 16-years-old, and from the Wechsler Adult Intelligence Scale (version IV) (3) to

the 17-year-old participants. Four subtests covering verbal comprehension (Vocabulary and either Similarities or Comprehension) and visual spatial (Block Design and either Visual Puzzles or Object Assembly from the Wechsler Intelligence Scale for Children, version III) (4) domains were completed. A verbal comprehension scaled score was computed by averaging the two verbal subtest scaled scores and a visual spatial scaled score was computed by averaging the two visual spatial subtest scaled scores.

Pubertal stage in reference to sex assigned at birth was self-reported using the Personal Development Scale (PDS) (5) and Cronbach's alpha was 0.94 for the cisgender boys and 0.70 for the cisgender girls/GD AFAB. A mean was calculated from these items where higher scores represented more advanced pubertal development. Cisgender girls and GD AFAB were also asked whether they had started menstruating and all AFAB participants (n = 34) answered affirmatively. Participants were further asked about the regularity of their menstrual periods and a dichotomous menstrual cycle regularity variable was created to represent participants with regular cycles versus participants with irregular cycles. Note that all participants had begun puberty prior to the time of participation as evidenced by mean scores greater than 1 on the PDS. A mean score of 1 would indicate pubertal development had not started.

Participants completed a medication log of all medications they were currently taking, the dosage, and any side effects. Nine participants were taking one (n = 6) or two (n = 3) medications. A dichotomous variable was created representing those not taking any medication (including one participant who was taking Cold FX, a natural health product) versus those taking any medications.

The Youth Self-Report (YSR) is a 118-item self-report questionnaire designed for youth between the ages of 11 and 18 years to assess general psychopathology (6, 7, 8). T scores for the internalizing problems, externalizing problems, and total problems factors were used. Also, dichotomous variables for internalizing problems, externalizing problems, and total problems were calculated, representing whether the T scores were in the clinical range (>90th percentile) or not.

3.2 Statistical Analyses

Using SPSS version 27, group differences on continuous demographic variables were examined with one-way analyses of variance (ANOVA). In the presence of a significant omnibus effect, post hoc comparisons were conducted with least significant difference (LSD) tests. For categorical variables, chi-square analyses or Fisher's Exact Test (if the expected cell size was less than 5) were conducted with follow-up Bonferroni-corrected z-tests that compare column proportions in the presence of a significant omnibus effect. A two-tailed critical p-value of 0.05 was used. Correlations between each variable and the 171 inter-network functional connectivity pairs were also conducted using Bonferroni corrections for multiple comparisons (see Results for adjustments used).

3.3 Results: Group Differences

For all continuous variables, there were no extreme deviations from normality based on skewness and kurtosis values, which were less than |2|.

One-way ANOVAs for verbal comprehension index, visual spatial index, personal development score, and externalizing problems T score indicated no significant between-group differences (Table S2). There was a significant difference for the internalizing problems T score such that the GD AFAB group scored significantly higher than both the cisgender girls (mean difference [MD] = 9.11, SE = 3.57, p = 0.014) and cisgender boys (MD = 18.01, SE = 3.69, p < 0.001), and the

cisgender girls scored significantly higher than the cisgender boys (MD = 8.91, *SE* = 3.63, p = 0.018). There was also a significant difference for total problems *T* score such that the GD AFAB scored significantly higher than both the cisgender girls (MD = 6.35, *SE* = 3.11, p = 0.047) and cisgender boys (MD = 14.00, *SE* = 3.21, $p < 0.001$). The cisgender girls scored significantly higher than the cisgender boys (MD = 7.66, *SE* = 3.17, p = 0.020), which differed from Skorska et al. (1), where there was no significant difference between cisgender girls and cisgender boys.

A Chi-square test for ethnicity and Fisher's exact tests for education of mother, education of father, externalizing problems in the clinical range, and regularity of menstrual cycle indicated these variables were not related to group (Table S2). There was a significant association between parent marital status and group. The GD AFAB group was significantly more likely to have parents who were separated/widowed/divorced than married/cohabitating, the cisgender boys were significantly more likely to have parents who were married/cohabitating than separated/widowed/divorced, and there was no significant difference between the two categories related to marital status within cisgender girls. Medication use was also related to group. The GD AFAB participants were significantly more likely to be taking medication than not taking medication, whereas the cisgender boys and girls were significantly more likely to be not taking medication than taking medication, which is not surprising given mental health diagnoses were an exclusion criterion for cisgender participants. Variables representing whether internalizing problems and total problems were in the clinical range were both associated with group. For both, the GD AFAB group was significantly more likely to have a clinical flag than a non-clinical flag, the cisgender boys were significantly more likely to have a non-clinical flag than a clinical flag, and there was no significant difference between clinical or non-clinical flag within cisgender girls.

3.4 Results: Correlations with IC Pairs

The full correlation table can be found in the Excel file titled "SkorskaetalGDrsfrmicorrcov_n49.xlsx" (9). There were 174 significant correlations prior to correction. The largest significant positive correlation occurred between IC06-IC09 and regularity of menstrual cycle (r = 0.49, p = 0.003, n = 34) and the smallest significant positive correlation occurred between IC02-IC07 and marital status (r = 0.29, p = 0.050, n = 48). The largest significant negative correlation occurred between IC01-IC08 and ethnicity (r = -0.48, p = 0.000468, n = 49) and the smallest significant negative correlation occurred between IC01-IC09 and the Personal Development Scale score (r = -0.28, p = 0.050, n = 49). There were 17 demographic variables and 171 intra-component connectivity network pairs. We used two different Bonferroni corrections. One very conservative correction considering the 17 variables and 171 network pairs, which totaled 2907 tests. The new p-value for these 2907 tests is 0.05/2907 = 0.0000172. The second less conservative correction involved only the 171 network pairs and the new p-value is 0.05/171 = 0.000292. Using both corrections for multiple tests there were no significant correlations between any of the demographic variables and the network pairs, including demographic variables that showed significant group differences.

4 Association of Resting-State Networks From Independent Component Analysis with Find Lab Atlas

We used the Find Lab atlas (10) to examine whether the resting-state networks (RSNs) derived from the group independent component analysis (ICA) reflect previously reported RSNs. Specifically, independent component (IC) spatial maps from the group ICA were correlated with the Find Lab atlas downloaded from https://findlab.stanford.edu/functional_ROIs.html using FSL's

(FMRIB Software Library, version 6.0.4, https://fsl.fmrib.ox.ac.uk/fsl/fslwiki/) fslcc and a correlation threshold of 0.20. Each IC/RSN could correlate with multiple Find Lab RSNs, although we found that apart from IC01, each IC/RSN only correlated with one Find Lab RSN. Correlations ranged from small (less than 0.29) to large (greater than 0.50) (Table S3). Thus, we were generally successful at replicating existing RSNs in our data. Figure 1 shows the RSNs in the current study.

5 Supplementary Tables

Table S1. Descriptive statistics for demographic and psychosexual variables of interest.

	Cisgender Boys	GD AFAB	Cisgender Girls	*F* (*df*)	*p*
n	15	17	17		
Age (months)					
M	184.73	192.00	191.71	0.68 (2, 46)	0.511
SD	24.69	14.87	19.00		
Range	147-216	162-216	152-214		
GIDYQ-AA					
M	4.91	2.19	4.90	743.35 (2, 46)[c]	< 0.001
SD	0.12	0.35	0.15		
Range (1-5)[a]	4.63-5.00	1.74-3.04	4.48-5.00		
Strength of attractions					
M	3.20	3.07	2.83	0.46 (2, 46)[d]	0.634
SD	1.25	1.29	0.76		
Range (1.41-7.07)[b]	1.70-5.37	1.41-6.05	1.41-4.44		
Degree of androphilia-gynephilia					
M	64.72	46.94	30.94	28.01 (2, 46)	< 0.001
SD	8.96	14.92	13.18		
Range (11-79)[b]	43.96-75.55	17.35-75.55	14.04-68.20		

Note. GIDYQ-AA = Gender Identity/Gender Dysphoria Questionnaire for Adolescents and Adults.
[a]Absolute range.
[b]Possible range for magnitude and phase (θ) based on EROS scores with absolute range of 1 to 5.
[c]A significant Levene's test ($p = 0.008$) warrants reporting of the robust tests of equality of means via the Welch test statistic (2, 28.86) = 463.34, $p < 0.001$, and the Brown-Forsythe test statistic (2, 26.07) = 778.65, $p < 0.001$.
[d]A significant Levene's test ($p = 0.039$) warrants reporting of the robust tests of equality of means via the Welch test statistic (2, 27.85) = 0.59, $p = 0.564$, and the Brown-Forsythe test statistic (2, 38.94) = 0.45, $p = 0.638$.

Table S2. Descriptive statistics for additional demographic variables.

	Cisgender Boys	GD AFAB	Cisgender Girls	*F* (*df*), χ^2 (*df*), or Fisher's Exact Test	*p*
n[a]	15	17	17		
Ethnicity, *n* (%)					
"European"/"White"	7 (46.7)	11 (64.7)	11 (64.7)	1.40 (2)	0.496
Other	8 (53.3)	6 (35.3)	6 (35.3)		
Mother's education, *n* (%)					
High school or less	0	4 (23.5)	1 (5.9)	4.02	0.112
Any post-secondary	13 (86.7)	12 (70.6)	14 (82.4)		
n	13	16	15		
Father's education, *n* (%)					
High school or less	1 (6.7)	6 (35.3)	3 (17.6)	3.46	0.201
Any post-secondary	11 (73.3)	9 (52.9)	11 (64.7)		
n	12	15	14		
Parent's marital status, *n* (%)					
Married/living together	14 (93.3)	7 (41.2)	15 (88.2)	15.60	< 0.001
Other	0	10 (58.8)	2 (11.8)		
n	14	17	17		
Verbal Comprehension					
M	13.00	11.74	11.91	1.41 (2, 45)	0.328
SD	2.34	2.27	2.94		
Range (1-19)[b]	9-17.50	6.5-15	5-17		
n	15	17	16		
Visual Spatial					
M	11.67	10.53	11.22	0.80 (2, 45)	0.456
SD	2.27	2.96	2.38		
Range (1-19)[b]	7.5-15.5	4-15	7.5-16.5		
n	15	17	16		
Personal Development Scale					
M	2.90	3.26	3.13	1.37 (2, 46)	0.265
SD	0.77	0.58	0.53		
Range (1-4)[b]	1.20-4.00	2.00-4.00	2.25-4.00		
Menstrual Cycle Regularity, *n* (%)					
Regular	-	15 (88.2)	10 (58.8)	-	0.118
Irregular	-	2 (11.8)	7 (41.2)		
Medication Use, *n* (%)					
None	15 (100)	8 (47.1)	17 (100)	18.33	< 0.001
1-2	0	9 (52.9)	0		
YSR: Internalizing Problems *T* Score					
M	48.80	66.81	57.71	11.94 (2, 45)	< 0.001
SD	10.48	11.77	8.38		
Range (27 or 30-100)[b,c]	35-66	49-90	45-80		
n	15	16	17		

	Cisgender Boys	GD AFAB	Cisgender Girls	*F* (*df*), χ^2 (*df*), or Fisher's Exact Test	*p*
YSR: Internalizing Problems Clinical Range, *n* (%)					
Non-clinical	14 (93.3)	6 (35.3)	13 (76.5)	11.46	0.003
Clinical	1 (6.7)	10 (58.8)	4 (23.5)		
YSR: Externalizing Problems *T* Score					
M	51.60	58.56	54.47	2.13 (2, 45)	0.131
SD	9.28	9.51	9.59		
Range (29-100)[b,c]	30-63	38-78	34-71		
n	15	16	17		
YSR: Externalizing Problems Clinical Range, *n* (%)					
Non-clinical	15 (100)	12 (70.6)	15 (88.2)	4.12	0.101
Clinical	0	4 (23.5)	2 (11.8)		
n	15	16	17		
YSR: Total Problems *T* Score					
M	49.93	63.94	57.59	9.52 (2, 45)	< 0.001
SD	8.64	9.13	9.00		
Range (26-100)[b,c]	32-62	49-81	42-76		
n	15	16	17		
YSR: Total Problems Clinical Range, *n* (%)					
Non-clinical	15 (100)	6 (35.3)	13 (76.5)	15.31	< 0.001
Clinical	0	10 (58.8)	4 (23.5)		
n	15	16	17		

Note. YSR = Youth Self-Report.

[a]This is the full sample size for each group. If there were missing data, the sample size is indicated with the relevant variable.

[b]Indicates absolute range.

[c]A *T* score above 63 is considered to be in the clinical range. For Internalizing *T* scores, the lower bound *T* score is 27 for birth-assigned females and the lower bound *T* score is 30 for birth-assigned males.

Table S3. Associations between the Find Lab atlas and independent components (ICs).

IC#	Network label/name	Correlation with the Find Lab atlas
IC01	Dorsal default mode network (DMN)	0.284
	Ventral DMN	0.350
IC02	Right executive control network	0.491
IC03	Language network	0.357
IC04	Left executive control network	0.465
IC05	High visual network	0.243
IC06	Ventral DMN	0.421
IC07	Dorsal DMN	0.507
IC08	Posterior salience network	0.287
IC09	Precuneus network	0.551
IC10	Primary visual network	0.460
IC11	Anterior salience network	0.503
IC13	Visuospatial network	0.258
IC14	Visuospatial network	0.373
IC15	High visual network	0.444
IC16	Sensorimotor network	0.282
IC17	Sensorimotor network	0.217
IC18	Auditory network	0.329
IC20	Basal ganglia network	0.336
IC21	Cerebellum	n/a (not in the Find Lab atlas)

6 Supplementary Figures

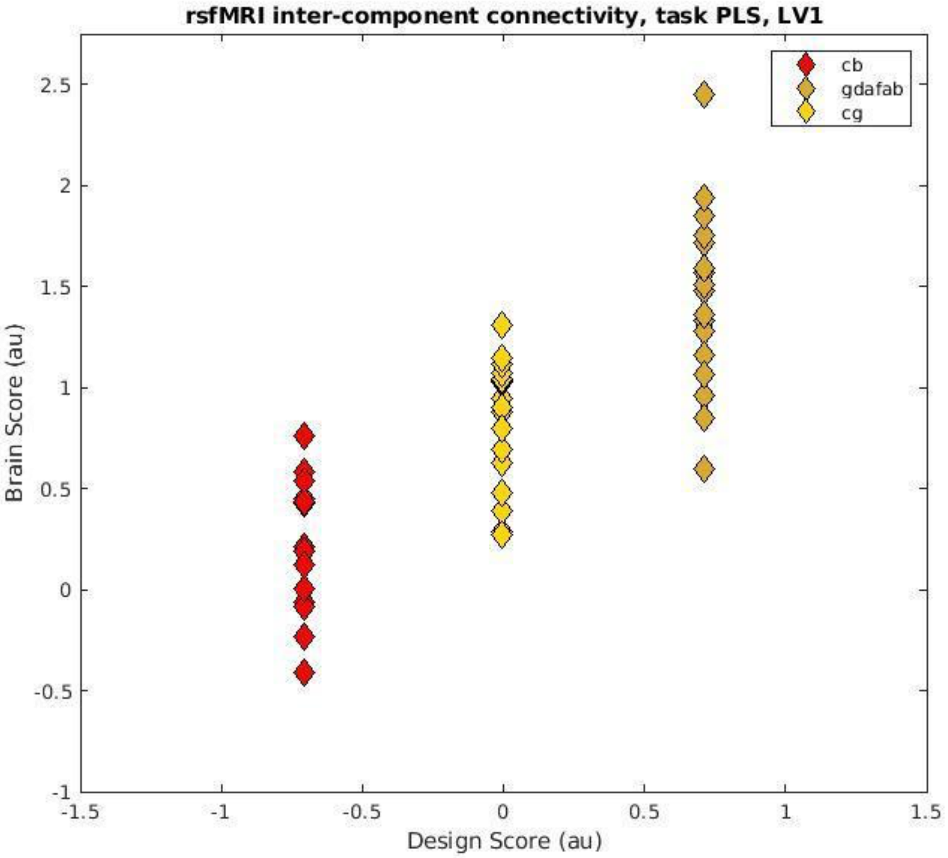

Supplementary Figure 1. The first PLS analysis results (brain scores by design scores). Brain scores plotted as a function of design scores for cisgender boys (red), cisgender girls (yellow), and GD AFAB (beige). These scores are also shown in Fig. 3, Panel A as bar plots with 95% CIs.

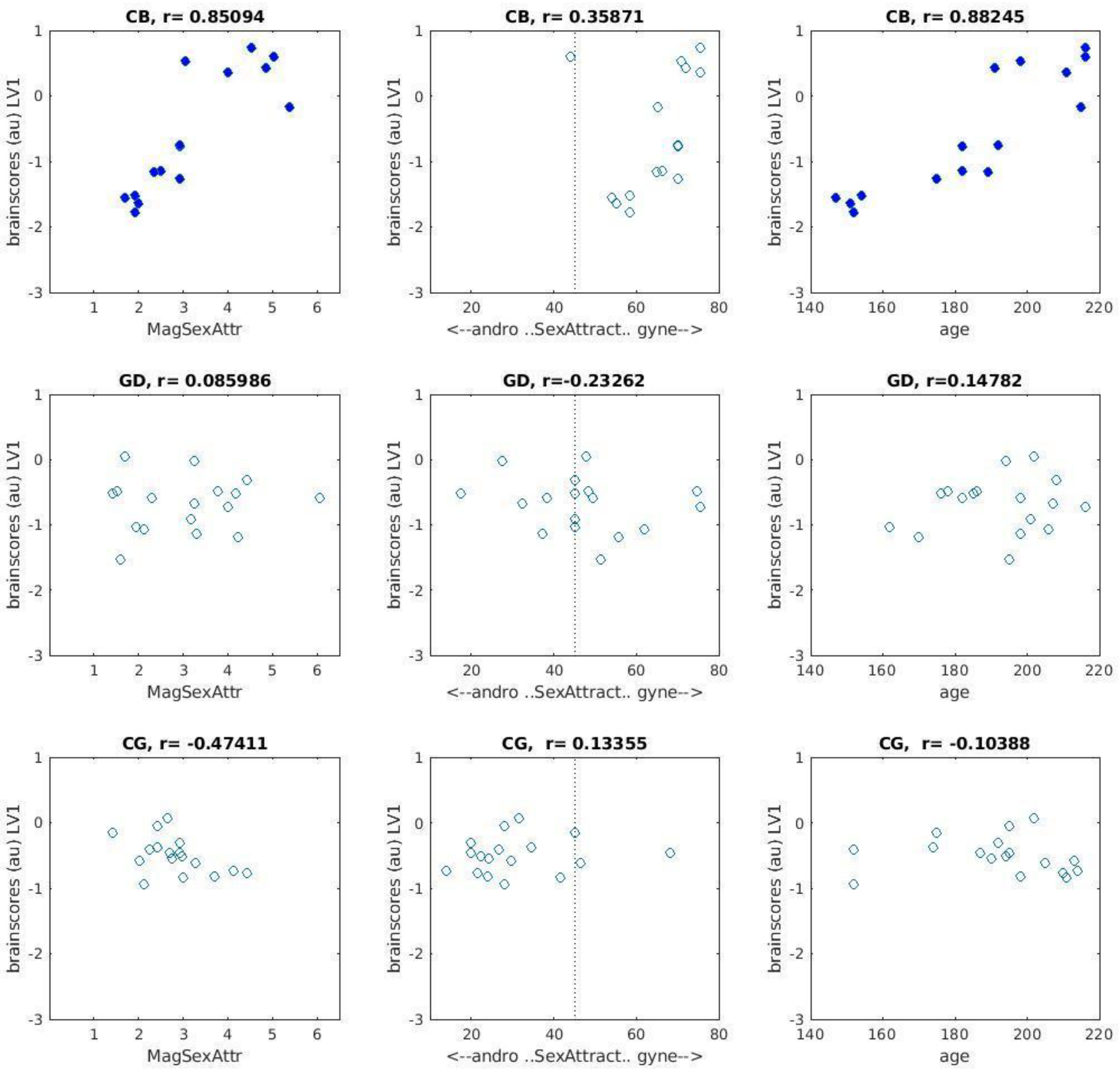

Supplementary Figure 2. Scatterplots showing the brain score-behavior correlations for all groups and behavioral measures for the first latent variable. These correlations are also shown in Fig. 4, Panel A as bar plots with 95% CIs. Left column is the strength of sexual attractions (MagSexAttr). Center column is the direction of sexual attraction (SexAttr) with a range from exclusively androphilic (11°) to exclusively gynephilic (79°). The dotted line at 45°indicates ambiphilic or asexual and the range is indicated by the dot-dashed line. Right column is age (months). The strength of the brain-behavior correlation is indicated at the top of each plot, and stable correlations are indicated by filled markers. CB = cisgender boys, GD = GD AFAB, CG = cisgender girls.

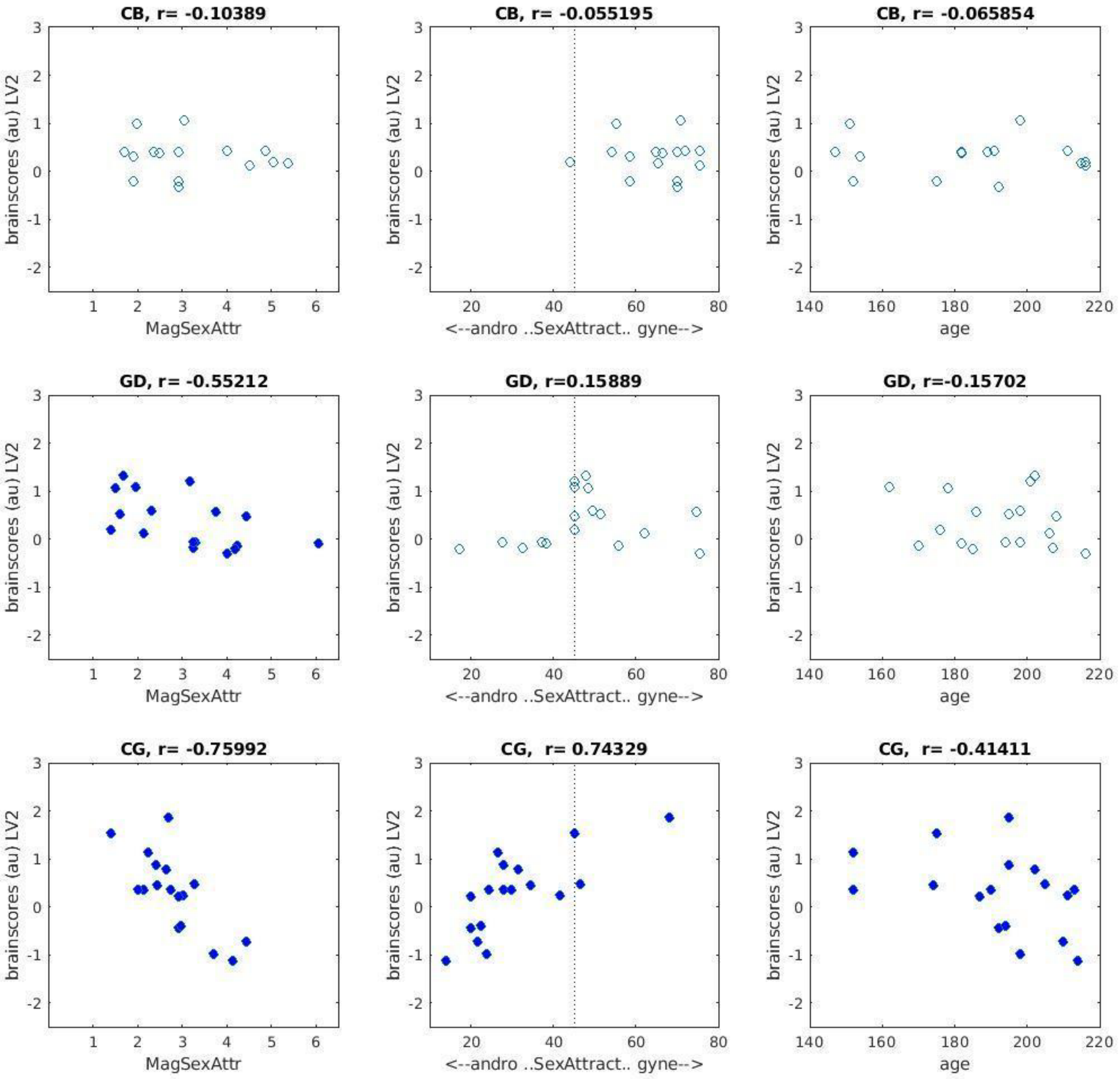

Supplementary Figure 3. Scatterplots showing the brain score-behavior correlations for all groups and behavioral measures for the second latent variable. These correlations are also shown in Fig. 5, Panel A as bar plots with 95% CIs. Left column is the strength of sexual attractions (MagSexAttr). Center column is the direction of sexual attraction (SexAttr) with a range from exclusively androphilic (11°) to exclusively gynephilic (79°). The dotted line at 45°indicates ambiphilic or asexual and the range is indicated by the dot-dashed line. Right column is age (months). The strength of the brain-behavior correlation is indicated at the top of each plot, and stable correlations are indicated by filled markers. CB = cisgender boys, GD = GD AFAB, CG = cisgender girls.

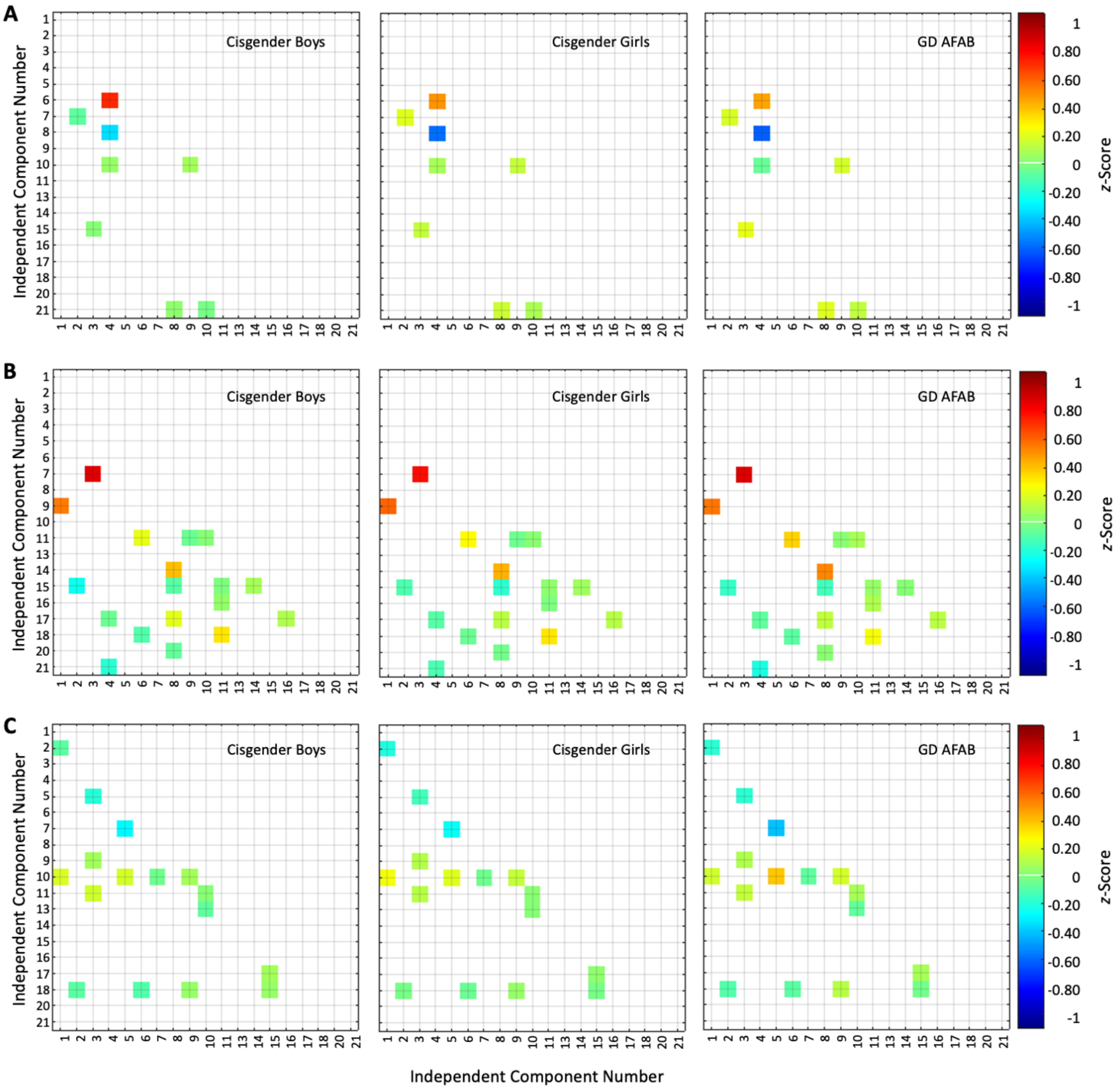

Supplementary Figure 4. Heat maps of *z*-scores within each group of the most stable saliences from each PLS. Panel A. Heat maps from the first PLS analysis. See Fig. 3 for the results of the first PLS analysis. **Panel B.** Heat maps for latent variable 1 from the second PLS analysis. See Fig. 4 for latent variable 1 results from the second PLS analysis. **Panel C.** Heat maps for latent variable 2 from the second PLS analysis. See Fig. 5 for latent variable 2 results from the second PLS analysis. The most stable saliences for 19 independent component (IC) pairs are shown. ICs 12, 19, and 22-25 were discarded as noise or non-gray matter signal. Network names are in Table 1.

7 References

1. Skorska MN, Chavez S, Devenyi GA, Patel R, Thurston LT, Lai M-C, et al. A multi-modal MRI analysis of cortical structure in relation to gender dysphoria, sexual orientation, and age in adolescents. Journal of Clinical Medicine (2021). 10:345. doi:10.3390/jcm10020345
2. Wechsler D. Wechsler intelligence scale for children (Fifth ed.). San Antonio, TX: The Psychological Corporation (2014).
3. Wechsler D. Wechsler adult intelligence scale (Fourth ed.). San Antonio, TX: Pearson (2008).
4. Wechsler D. Wechsler intelligence scale for children (Third ed.). San Antonio, TX: The Psychological Corporation (1991).
5. Petersen AC, Crockett L, Richards M, Boxer AA. Self-report measure of pubertal status: Reliability, validity, and initial norms. J. Youth Adolesc. (1988) 17:117–133. doi:10.1007/BF01537962
6. Achenbach TM. Manual for the Youth Self-Report and 1991 Profile. Burlington, VT: University of Vermont Department of Psychiatry (1991).
7. Achenbach TM, Edelbrock C. Manual for the Youth Self-Report and Profile. Burlington, VT: University of Vermont Department of Psychiatry (1986).
8. Achenbach TM, Rescorla LA. Manual for the ASEBA School-Age Forms & Profiles. Burlington, VT: University of Vermont, Research Center for Children, Youth, & Families (2001).
9. Skorska MN, Lobaugh NJ, Lombardo MV, van Bruggen N, Chavez S, Thurston LT, et al. Replication data for: Inter-network brain functional connectivity in adolescents assigned female at birth who experience gender dysphoria. Borealis, the Canadian Dataverse Repository (2022). doi:10.5683/SP3/VQG2X6
10. Shirer WR, Ryali S, Rykhlevskaia E, Menon V, Greicius MD. Decoding subject-driven cognitive states with whole-brain connectivity patterns. Cerebral Cortex (2012) 22:158-165. doi:10.1093/cercor/bhr099
